# Itaconate Production from Crude Substrates with *U. maydis*: Scale-up of an Industrially Relevant Bioprocess

**DOI:** 10.1186/s12934-024-02295-3

**Published:** 2024-01-20

**Authors:** Tabea Helm, Thilo Stausberg, Martina Previati, Philipp Ernst, Bianca Klein, Tobias Busche, Jörn Kalinowski, Daniel Wibberg, Wolfgang Wiechert, Lien Claerhout, Nick Wierckx, Stephan Noack

**Affiliations:** 1 Institute of Bio- and Geosciences - IBG-1: Biotechnology, Forschungszentrum Jülich GmbH, D-52425 Jülich, Germany; 2 Bio Base Europe Pilot Plant vzw, Ghent, B-9042 Belgium; 3 Medical School East Westphalia-Lippe & Center for Biotechnology (CeBiTec), Bielefeld University, Bielefeld, Germany; 4 Center for Biotechnology (CeBiTec), Bielefeld University, D-33615 Bielefeld, Germany

**Keywords:** *Ustilago maydis*, Itaconate, Industrial waste streams, Molasses, Glycerol, Scale-up

## Abstract

**Background:**

Industrial by-products accrue in most agricultural or food-related production processes, but additional value chains have already been established for many of them. Crude glycerol has a 60% lower market value than commercial glucose, as large quantities are produced in the biodiesel industry, but its valorisation is still underutilized. Due to its high carbon content and the natural ability of many microorganisms to metabolise it, microbial upcycling is a suitable option for this waste product.

**Results:**

In this work, the use of crude glycerol for the production of the value-added compound itaconate is demonstrated using the smut fungus *Ustilago maydis*. Starting with a highly engineered strain, itaconate production from an industrial glycerol waste stream was quickly established on a small scale, and the resulting yields were already competitive with processes using commercial sugars. Adaptive laboratory evolution resulted in an evolved strain with a 72% increased growth rate on glycerol. In the subsequent development and optimisation of a fed-batch process on a 1.5-2 L scale, the use of molasses, a side stream of sugar beet processing, eliminated the need for other expensive media components such as nitrogen or vitamins for biomass growth. The optimised process was scaled up to 150 L, achieving an overall titre of 72 g L^− 1^, a yield of 0.34 g g^− 1^, and a productivity of 0.54 g L^− 1^ h^− 1^.

**Conclusions:**

Pilot-scale itaconate production from the complementary waste streams molasses and glycerol has been successfully established. In addition to achieving competitive performance indicators, the proposed dual feedstock strategy offers lower process costs and carbon footprint for the production of bio-based itaconate.

**Supplementary Information:**

The online version contains supplementary material available at 10.1186/s12934-024-02295-3.

## Background

Refined glycerol has a wide array of uses, for example as a humectant in the food- and cosmetic industry or in the explosives industry in the form of nitro-glycerine [1]. However, crude glycerol derived from the biodiesel industry contains various impurities, making it unsuitable for most common glycerol applications and resulting in its classification as a waste product [2]. Since the purification of glycerol is costly, alternative drains for the large amounts of the unrefined by-product are needed. Today, the two major uses of crude glycerol are in animal feed and as an additive in cement [3, 4]. Nonetheless, the accumulation of crude glycerol still greatly exceeds the demand, which is reflected in its extraordinarily low market price. Since the advent of biodiesel production, the price for crude glycerol dropped from US$ 0.25 to US$ 0.04–0.09 per pound [5, 6]. Thus, because of its potential to decrease process costs and also increase the sustainability of processes, the waste product has received considerable attention as a potential substrate for new applications, for example in the biotechnology sector where it is studied as a potential feedstock for the fermentative production of value-added chemicals.

Successful fermentation for the production of 1,3-propanediol with *Klebsiella pneumonia* [7] or PHB with *Zobellella denitrificans* [8] have demonstrated that crude glycerol can be efficiently metabolised by different microorganisms and therefore reduce the cost of the process greatly compared to using commercial sugars as the sole carbon source. Another study could demonstrate that *Yarrowia lipolytica* produces citric acid from glycerol at the same yield that is typically achieved from fermentations with yeasts on commercial sugars [9].

Like most industrial by-products, crude glycerol comes with a set of challenges when applied as a substrate in biotechnological processes. Possibly the most acute challenge when working with crude glycerol is the aforementioned impurities contained in the unrefined substrate. Among other factors, the catalyst used, the transesterification efficiency, and the recovery efficiency of the biodiesel impose the kind and concentration of impurities present in crude glycerol. Common impurities include high amounts of salts, methanol, and fatty acids [10]. All these components can interfere negatively with the metabolism or integrity of microorganisms [11]. Another noteworthy challenge with these processes is that some microorganisms were not optimised for the growth and production on glycerol. In these organisms, genetic engineering has not focused on the respective genes that are relevant for efficient growth on glycerol. A study by Zambanini *et al.* tackled the issue of slow conversion rates by applying adaptive laboratory evolution to their producing organism *Ustilago vetiveriae* to optimise its growth on glycerol, which resulted in an increased growth rate and itaconate production [12].

Itaconate, a dicarboxylic acid, is used as a building block for the production of a wide range of products, including plastics, resins, and fragrances [13, 14]. The traditional production of itaconate involves the producer strain *Aspergillus terreus*, which due to its pathogenic nature, is highly regulated within the EU [15]. In fermentations using *A. terreus* with glucose as the substrate, titres of 160 g L^− 1^ with a yield of 0.56 g g^− 1^ can be reached [16]. However, previous studies could demonstrate advantages of using the Basidiomycete *Ustilago* as it exhibits yeast-like growth and is therefore easier to handle in the lab and in production settings. Competitive yields and titres could be reached using several species of the genus *Ustilago* on different carbon sources (Table 1).

**Table 1:** Key performance indicators of different itaconate producer strains using *Ustilago spp*

Strain	Cultivation method, carbon source	Titre [g L^-1^]	Yield [g g^-1^]	Productivity [g L^-1^ h^-1^]	Reference
*U. maydis*	1 L STR, high-density fed-batch, glucose	74.9	0.54	0.53	[17]
*U. cynodontis*	0.5 L STR, high-density pulsed fed-batch, glucose	83.0	0.30	0.59	[18]
*U. vetiveriae*	24-well plate, glycerol	34.7	0.18	0.09	[12]
*U. maydis and Trichoderma reesei*	1 L shake flasks, cellulose	33.8	0.16	0.07	[19]
*U. maydis*	250 mL shake flasks, brewer’s spent grain	6.0	0.38	0.11	[20]
*U. cynodontis*	100 L STR batch, thick juice	66.4	0.44	0.35	[21]

In this study we used the Basidiomycete *Ustilago maydis* for the production of itaconate from the waste stream crude glycerol. While competitive itaconate titres and yields were quickly achieved with an engineered producer strain, the productivity of the process was rather low. Therefore, we performed adaptive laboratory evolution with the producer strain to increase its glycerol conversion capacity. As a second strategy, molasses was supplied as a source of minerals and vitamins to enhance the production performance, and this setup was successfully scaled up to a 150 L fed-batch fermentation process.

## Results and discussion

Using the industrial waste product crude glycerol as the carbon source in biotechnological production processes has the potential to increase their sustainability significantly. Therefore we explored the feasibility of applying crude glycerol to the production of itaconate using the fungus strain *U. maydis* MB215 ∆*cyp3* ∆MEL ∆UA ∆*dgat* ∆P_*ria1*_::P_*etef*_ ∆*fuz7* P_*etef*_*mttA,* which has been engineered for efficient itaconate production from glucose [22]. In prior work we have demonstrated that the strain can efficiently produce itaconate from industrial side streams, such as molasses, with a high tolerance for impurities [23].

### Phenotyping on pure and crude glycerol at 1 mL scale

When working with an industrial by-product as the feedstock for biotechnological applications, impurities in the substrate are part of the most crucial challenges. The crude glycerol used in this study stems from a biodiesel manufacturing plant, which stated a purity of more than 80% with the main other components being water and salts like NaCl. Moreover, our GC-ToF-MS analysis revealed no contamination of methanol or other organic compounds (data not shown).

**Fig. 1:**
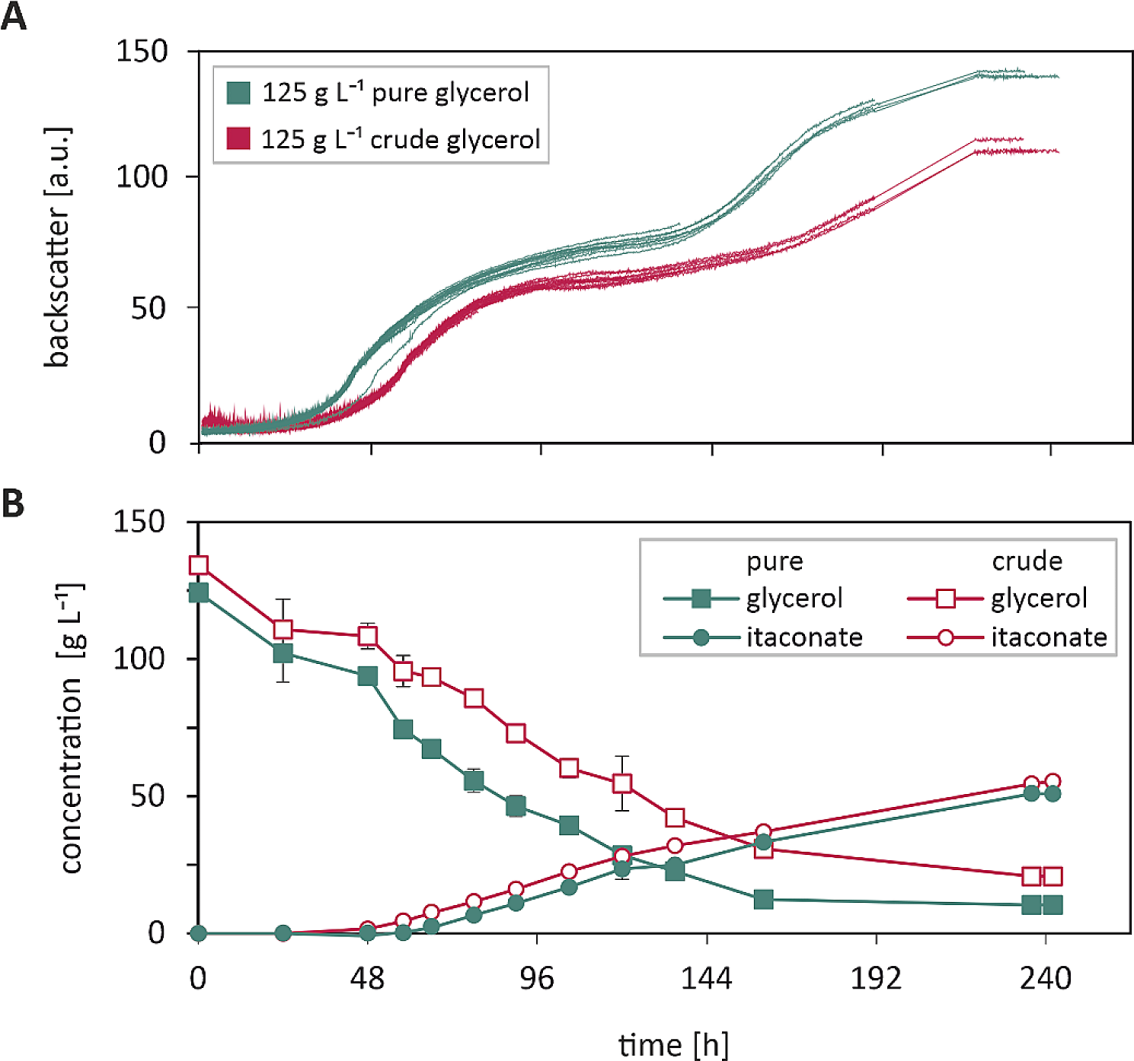
Phenotyping on pure and crude glycerol at 1 mL scale. Strain *U. maydis* MB215 ∆*cyp3* ∆MEL ∆UA ∆*dgat* ∆P_*ria1*_::P_*etef*_ ∆*fuz7* P_*etef*_*mttA* was grown in defined MTM with 125 g L^-1^ crude or pure glycerol as the sole carbon source and 2.4 g L^-1^ NH_4_Cl. The cultivation was performed in a BioLector Pro under the following conditions: 1400 rpm, 30 °C, 85% humidity, online measurements were taken every 10 min, sacrifice samples were taken in irregular intervals during the cultivation. Metabolites were analysed in duplicate via HPLC

In order to explore the effects of the contained salts on the growth and the product formation of the *U. maydis* strain, 125 g L^− 1^ pure or crude glycerol, respectively, were supplied to defined MTM medium and the performance of the strain was compared between the two media (Fig. 1). Under the pure as well as crude glycerol condition, the strain was able to metabolise the supplied carbon source and produce biomass, albeit both happened slightly more efficient for the pure glycerol condition. Notably, when only 50 g L^− 1^ glycerol was supplied, no difference in the growth behaviour between the conditions was visible (cf. Supplemental Figure S1), indicating that the impurities only have an effect when crude glycerol is supplied in higher concentrations. Regarding itaconate production, no significant difference could be observed between the two conditions (Table 2). The yield at which itaconate was produced was calculated to be 0.49 g g^− 1^ and 0.45 g g^− 1^ for the pure and crude condition, respectively. It can therefore be concluded that the effect of the impurities contained in the crude glycerol used for this study are minor. Under the same cultivation conditions we previously reported a yield of 0.33 g g^− 1^ using glucose as the sole carbon source [23].

**Table 2:** Key performance indicators of *U. maydis* itaconate producer strains. KPIs from batch and fed-batch cultivation experiments with the engineered (ori) and additionally evolved (evo) strain are derived either directly from measurements or via process modelling (1.5 L fed-batch only). All values represent time-dependent maxima, and the referenced phase (b = batch, f = feed, o = overall) is given in brackets

Process	Batch (1 mL)		Fed-batch (1.5 L)		Fed-batch (2 L)	Fed-batch (150 L)
*U. maydis* strain	Ori		Ori	Evo	Ori	Ori
Condition	Pure glycerol	Crude glycerol	Crude glycerol	Crude glycerol	Molasses (b) Crude glycerol (f)	Molasses (b) Crude glycerol (f)
Itaconate titre [g L^− 1^]	55.43	51.14	74.48	75.54	90.93	72.04
Itaconate yield [g g^− 1^]	0.49	0.45	0.328 (b) 0.669 (f)	0.253 (b) 0.512 (f)	0.33 (o)	0.34 (o)
Specific growth rate [h^− 1^]	-	-	0.053 (b) 0.0 (f)	0.091 (b) 0.0 (f)	-	-
Glycerol uptake rate [g g_CDW_ h^− 1^]	-	-	0.065 (b) 0.044 (f)	0.096 (b) 0.048 (f)	-	-
Itaconate production rate [g g_CDW_ h^− 1^]	-	-	0.031 (b) 0.029 (f)	0.036 (b) 0.025 (f)	-	-
Itaconate productivity [g L^− 1^ h^− 1^]	0.23 (o)	0.22 (o)	0.16 (o)	0.17 (o)	0.44 (o)	0.54 (o)

While this shows the great potential of crude glycerol as an alternative carbon source, the rates at which the conversion happens are significantly lower compared to previous experiments performed on commercial sugars. When grown on 100 g L^− 1^ glucose under otherwise similar conditions, *U. maydis* was able to consume most of the carbon source within the first 48 h, whereas it took the strain 96 h (pure) to 136 h (crude) to convert the same amount of glycerol.

### Proteomic study of glycerol catabolism in *U. maydis*

**Fig. 2:**
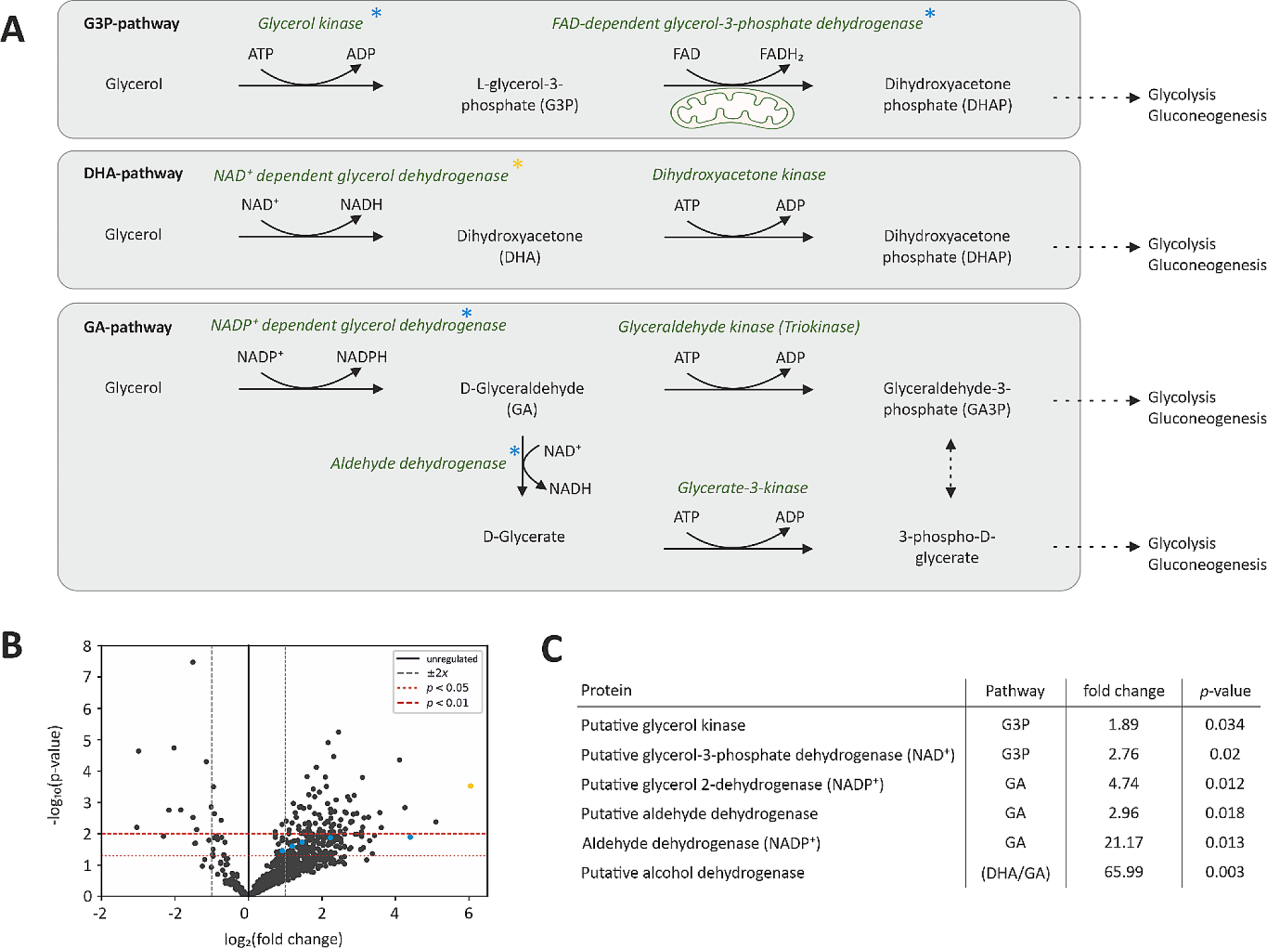
Proteomic studies of glycerol catabolism in *U. maydis*. (**A**) Glycerol degradation pathways as proposed for *S. cerevisiae*. Proteins identified in *U. maydis* MB215 ∆*cyp3* ∆MEL ∆UA ∆*dgat* ∆P_*ria1*_::P_*etef*_ ∆*fuz7* P_*etef*_*mttA* are highlighted with an asterisk. (**B**) Volcano plot of all proteins detected in a sample of *U. maydis* and their differential expression in glycerol-containing medium compared to glucose-containing medium. Fold changes describe the relative expression of the proteins between glycerol and glucose conditions, i.e. for log_2_-values > 1 (two-fold) and *p*-values < 0.05, significantly higher expressed proteins are found under the glycerol condition. (**C**) Expression levels of putative proteins involved in the glycerol catabolism in *U. maydis* as annotated in [26]

Previous studies have shown that fungi of the genus *Ustilago* are generally able to metabolise glycerol and produce valuable products, including itaconate [12, 24]. Nevertheless, the pathway with which *Ustilago spp*. metabolise glycerol has yet to be elucidated. Although a great diversity in the ability to utilise glycerol is observed among different yeast species, the studies performed on other fungus strains might be an indication of possible catabolic pathways. Previous studies have identified three possible pathways with which fungi metabolise glycerol (Fig. 2A): The phosphorylative glycerol catabolic pathway (G3P-pathway), the catabolic dihydroxyacetone (DHA) pathway, and the d-glyceraldehyde (GA) pathway. The first pathway is widespread among fungi and involves a glycerol kinase and an FAD-dependent glycerol-3-phosphate dehydrogenase. Glycerol is fed into glycolysis via the intermediate l-glycerol-3-phosphate (G3P). The second pathway starts with an NAD^+^-dependent glycerol dehydrogenase to oxidise glycerol to DHA, which is subsequently phosphorylated to dihydroxyacetone phosphate (DHAP). The third pathway connects the oxidation of glycerol to GA by an NADP^+^-dependent glycerol dehydrogenase (GDH) with the further conversion by either a glyceraldehyde kinase or an aldehyde dehydrogenase before it enters central carbon metabolism as a phosphorylated compound [25].

Proteome analysis of *U. maydis* cells grown in defined MTM with 50 g L^− 1^ glucose or glycerol revealed that both enzymes related to the G3P-pathway are present in *U. maydis* and show a higher expression in the cells grown on glycerol relative to those grown on glucose as the carbon and energy source (Fig. 2B). Additionally, some of the enzymes described in the GA-pathway were detected in *U. maydis*, of which the NADP^+^-dependent aldehyde dehydrogenase is expressed 21-fold higher under the glycerol condition (Fig. 2C). Another protein of interest is a putative alcohol dehydrogenase I with sequence identities of > 90% with alcohol dehydrogenases in other fungi species. The expression of this protein under glycerol conditions is 66-fold higher compared to the glucose condition (Fig. 2B, highlighted in yellow). Noteworthy, the structure of these zinc-containing alcohol dehydrogenases shows high similarities to those of glycerol dehydrogenases which are also known to use zinc as a cofactor [27, 28]. Some alcohol dehydrogenases have even been shown to function on glycerol as substrate [27], albeit at a low rate.

Overall, this data suggests that *U. maydis* has all prerequisites for the conversion of glycerol via the G3P-pathway but this is possibly not the only active pathway of the glycerol catabolism.

### Adaptive laboratory evolution to increase glycerol conversion

Considering the comparatively low conversion rates for glycerol, measures were taken to enhance the productivity of the process. The first measure included an optimisation of the producer strain through adaptive laboratory evolution (ALE) with the goal to improve the growth rate of the strain on glycerol and thus achieve an earlier onset of the itaconate production and an overall higher productivity of the process.

**Fig. 3:**
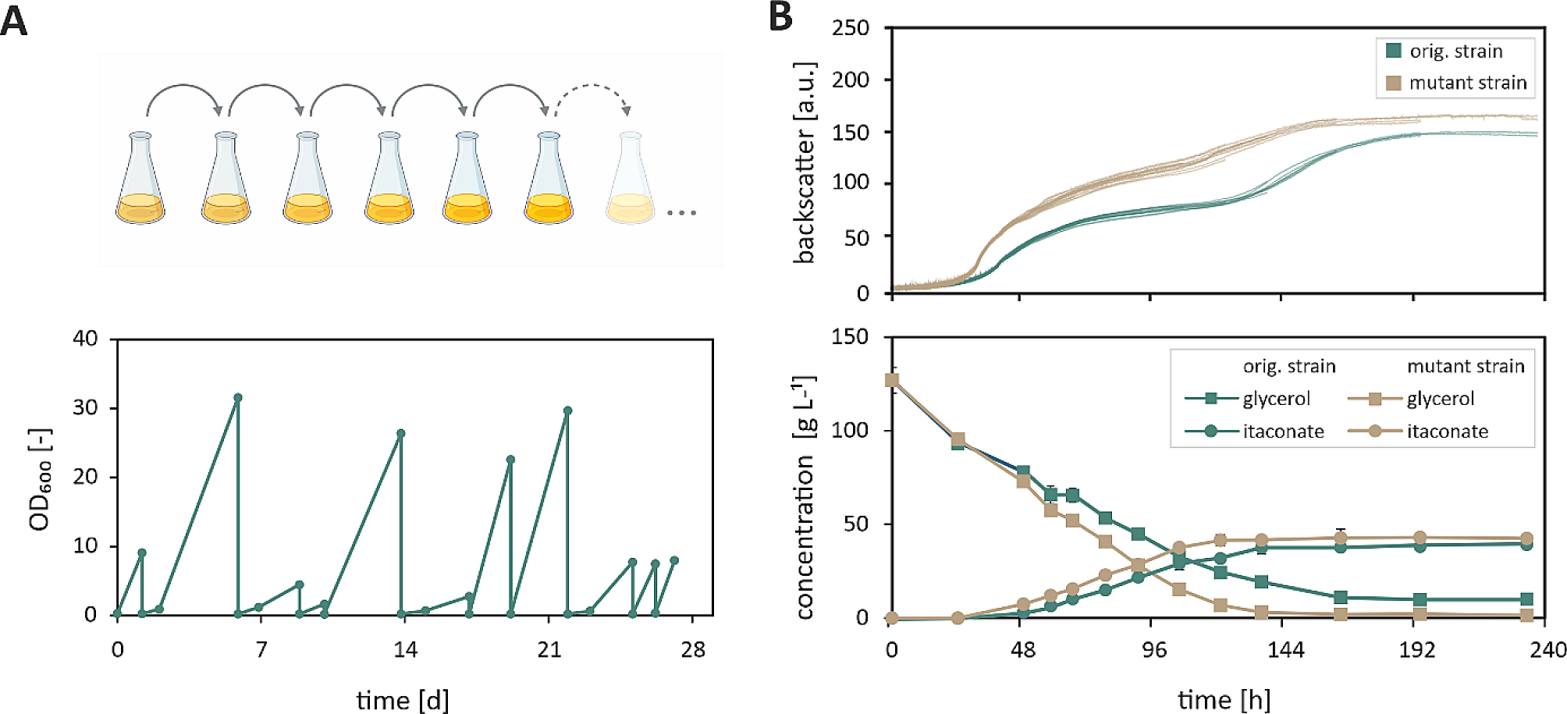
Adaptive laboratory evolution to increase glycerol conversion. (**A**) Inoculation scheme of a manual ALE in shake flasks. OD-values for a repetitive batch experiment (11 cycles) performed in shake flasks. *U. maydis* MB215 ∆*cyp3* ∆MEL ∆UA ∆*dgat* ∆P_*ria1*_::P_*etef*_ ∆*fuz7* P_*etef*_*mttA* was grown in defined MTM with 100 g L^-1^ pure glycerol as the sole carbon source and 2.4 g L^-1^ NH_4_Cl. (**B**) Verification experiment of the evolved strain compared to the original *U. maydis* strain in defined MTM containing 125 g L^-1^ pure glycerol and 2.4 g L^-1^ NH_4_Cl. The cultivation was performed in a BioLector Pro under the following conditions: 1400 rpm, 30 °C, 85% humidity, online measurements were taken every 10 min, sacrifice samples were taken in irregular intervals during the cultivation. Metabolites were analysed in duplicate via HPLC

For maximum efficiency and reproducibility, the experiment was initially planned in an automated fashion using a repetitive batch approach in microtiter plates, as previously described by Radek et al. [29]. However, we observed that the low growth rates of the eukaryotic strain are not compatible with this method, since persistent contamination with prokaryotic cells occurred and could not be contained within the semi-sterile environment of our robotic platform (Supplementary Figure S2). Hence, the ALE experiment was carried out in shake flasks filled with defined MTM containing 100 g L^− 1^ pure glycerol as the selection pressure. In total, 11 cycles were inoculated (Fig. 3A).

The success of the ALE was determined through a verification experiment. Both the original (*U. maydis* ori) and the evolved (*U. maydis* evo) strain were grown in defined MTM containing 125 g L^− 1^ pure glycerol (Fig. 3B). *U. maydis* evo showed an earlier onset of growth and thus a shortened lag phase. Additionally, growth to a higher cell density could be observed and, unlike the original strain, all glycerol was consumed.

A previous study reported a loss of itaconate production ability in *U. vetiveriae* following an ALE experiment on glycerol [12]. Under natural circumstances itaconate is hypothesised to be beneficial for *Ustilago spp.* by lowering the pH and hence giving the cells a competitive advantage over other organisms [30]. Under laboratory conditions and without competitors, the additional energy required for the production of itaconate could thus be a disadvantage, leading to the reduction or loss of the itaconate production through ALE. A loss of itaconate production ability was therefore circumvented by supplying sufficient amounts of the nitrogen source NH_4_Cl, as the synthesis of itaconate in *Ustilago spp.* only starts when no nitrogen is present in the medium. In accordance with our own previous study, the optimum C/N ratio for itaconate production under the present conditions is 74 mol_C_ mol_N_^−1^ [23], which was supplied in this experiment as well. However, since the re-inoculations happened while the cultures were still in the exponential phase, nitrogen was present in the medium at all times. Consequently, the itaconate production was not triggered before the inoculation of the next repetitive batch so that it has no influence on the fitness of the populations and is therefore not relevant as a selection pressure. With this experimental setup, comparable itaconate titres of 39.12 g L^− 1^ and 43.09 g L^− 1^ were obtained with *U. maydis* ori and *U. maydis* evo, respectively (Fig. 3B).

In an effort to decipher the genetic changes underlying the improved growth phenotype on glycerol of the evolved strain, whole genome sequencing was performed on *U. maydis* evo and *U. maydis* ori. In total, the sequencing data revealed 96 single nucleotide polymorphisms (SNP) in the evolved strain, of which 9 are located in encoding areas. Unfortunately, all 9 gene sequences are currently not identifiable through further bioinformatic analysis, such as a BLAST search. Alternatively, the mutations in intergenic regions could also have an impact on the glycerol metabolism or transport through regulatory effects. However, identifying specific regulatory areas for glycerol metabolism-related genes in eukaryotes is challenging and demands additional metabolic engineering work to be done.

We thus report that we have generated an evolved *U. maydis* itaconate producer strain with improved growth behaviour on glycerol, although the exact mechanism remains to be elucidated.

### Scale-up to 1.5 L bioreactor environment

**Fig. 4:**
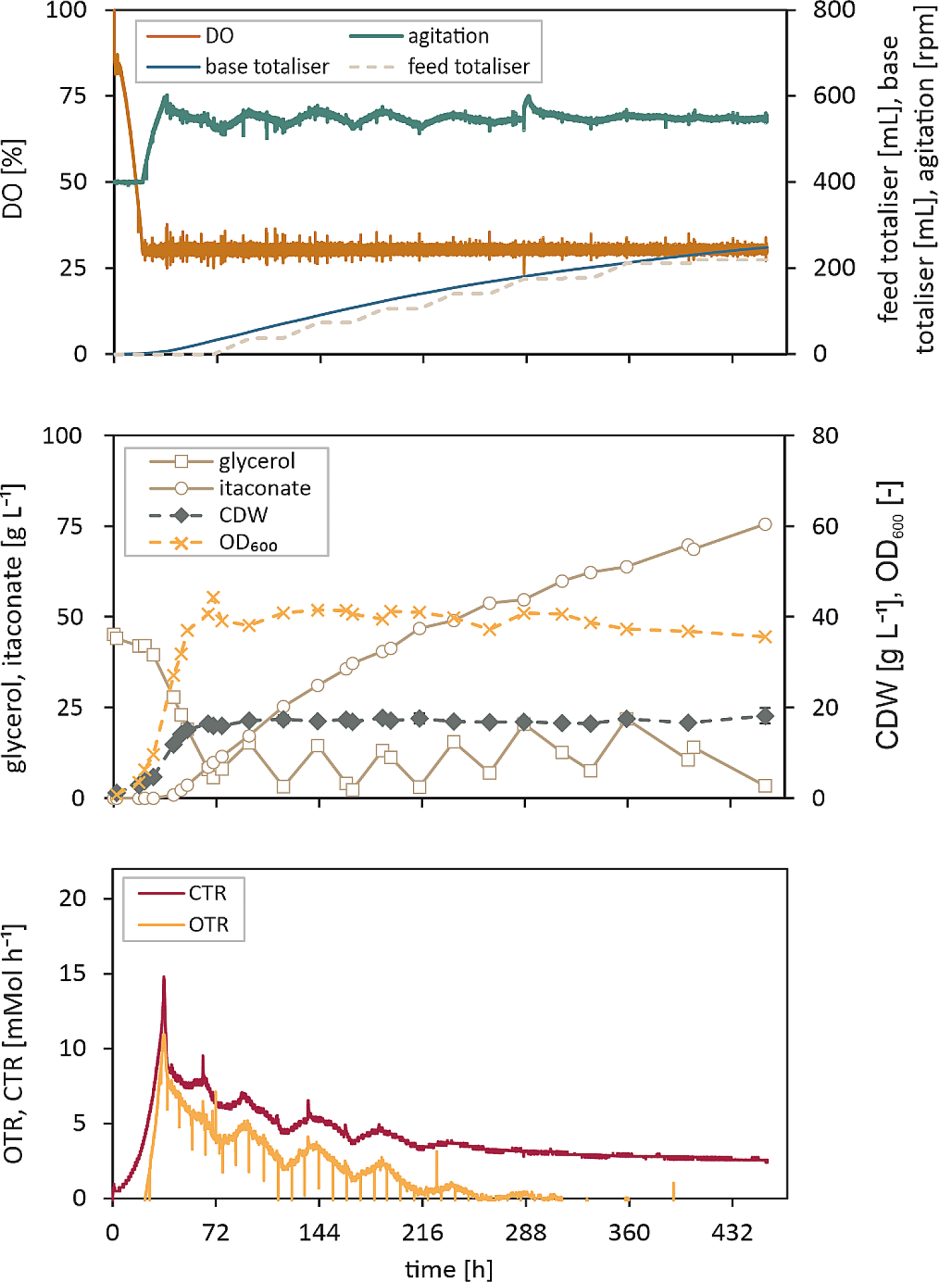
Scale-up to 1.5 L bioreactor environment. Fed-batch fermentation with *U. maydis* evo grown on crude glycerol as the sole carbon and energy source in the batch medium and in the feed. The cultivation was performed in a 1.5 L bioreactor with a filling volume of 0.8 L at 30 °C. Dissolved oxygen tension was kept > 30% and the pH was kept constant at 6.5. Samples were taken regularly and analysed via HPLC

In order to explore the scalability of the itaconate production process with both *U. maydis* ori and *U. maydis* evo, a 1.5 L fed-batch fermentation was performed using crude glycerol as the only source of carbon and energy in the batch medium as well as the feed (Fig. 4, Supplemental Figure S3).

Comparing the growth phases of both strains, the results for the batch phase match the previously shown results from the microscale experiments: The *U. maydis* evo strain showed a shortened lag phase on glycerol and the final cell density was again higher than that of *U. maydis* ori (cf. Figure 3B). The maximum specific growth rate determined by process modelling was increased by 72% to 0.091 h^− 1^ (Table 2). Consequently, the batch glycerol was depleted after 70 h by *U. maydis* evo compared to 116 h by *U. maydis* ori, providing a clear advantage for the evolved strain in terms of biomass production.

When all batch glycerol was consumed, feeding was started at the lowest possible rate of 1.5 mL h^− 1^ in both cases. Due to the comparatively low conversion rates during the growth-decoupled production phase, glycerol accumulated in the medium, so feeding was stopped after about 24 h until most of the glycerol was consumed, whereupon feeding was resumed. This feeding strategy proved to be quite beneficial for both strains, as respiratory activity increased each time the feed was started again.

While the biomass formation of *U. maydis* evo was significantly more efficient, *U. maydis* ori does perform better in the itaconate production phase. The latter started in both strains when reaching about two thirds of the maximum cell density, indicating the depletion of ammonia in the medium. After 108 h and 72 h, respectively, cell density remained almost constant in both processes until the end of the fermentation. With *U. maydis* ori, a yield of 0.67 g g^− 1^ was obtained in the feeding phase (cf. Table 2), which corresponds to 95% of the theoretical maximum of 0.706 g g^− 1^. Interestingly, *U. maydis* evo achieved only 73% of the maximum yield. One explanation for this could be an increased production of an unknown by-product, which was also included in the process model (see Supplementary Figure S5).

At the end of the fermentations, comparable titres of 74.48 g L^− 1^ and 75.54 g L^− 1^ were obtained for *U. maydis* ori and *U. maydis* evo, respectively, within the same total process time of 456 h. Finally, the higher conversion efficiency of *U. maydis* ori for glycerol to itaconate resulted in a slightly higher overall productivity of 0.17 g L^− 1^ h^− 1^ (Table 2).

### Supply of complementary feedstocks for batch and fed-batch operation

Since the improved growth rate of *U. maydis* evo on crude glycerol did not significantly increase overall productivity and itaconate yield was even lower, all further experiments were conducted with *U. maydis* ori. In order to make the process more efficient, the combined supply of complementary feedstocks for the biomass and itaconate production phases was investigated. As mentioned before, the feasibility of producing itaconate from molasses has been demonstrated in a previous study [23]. In this study, we reported that molasses contains many nitrogen sources that could promote growth but possibly hinder efficient itaconate production.

**Fig. 5:**
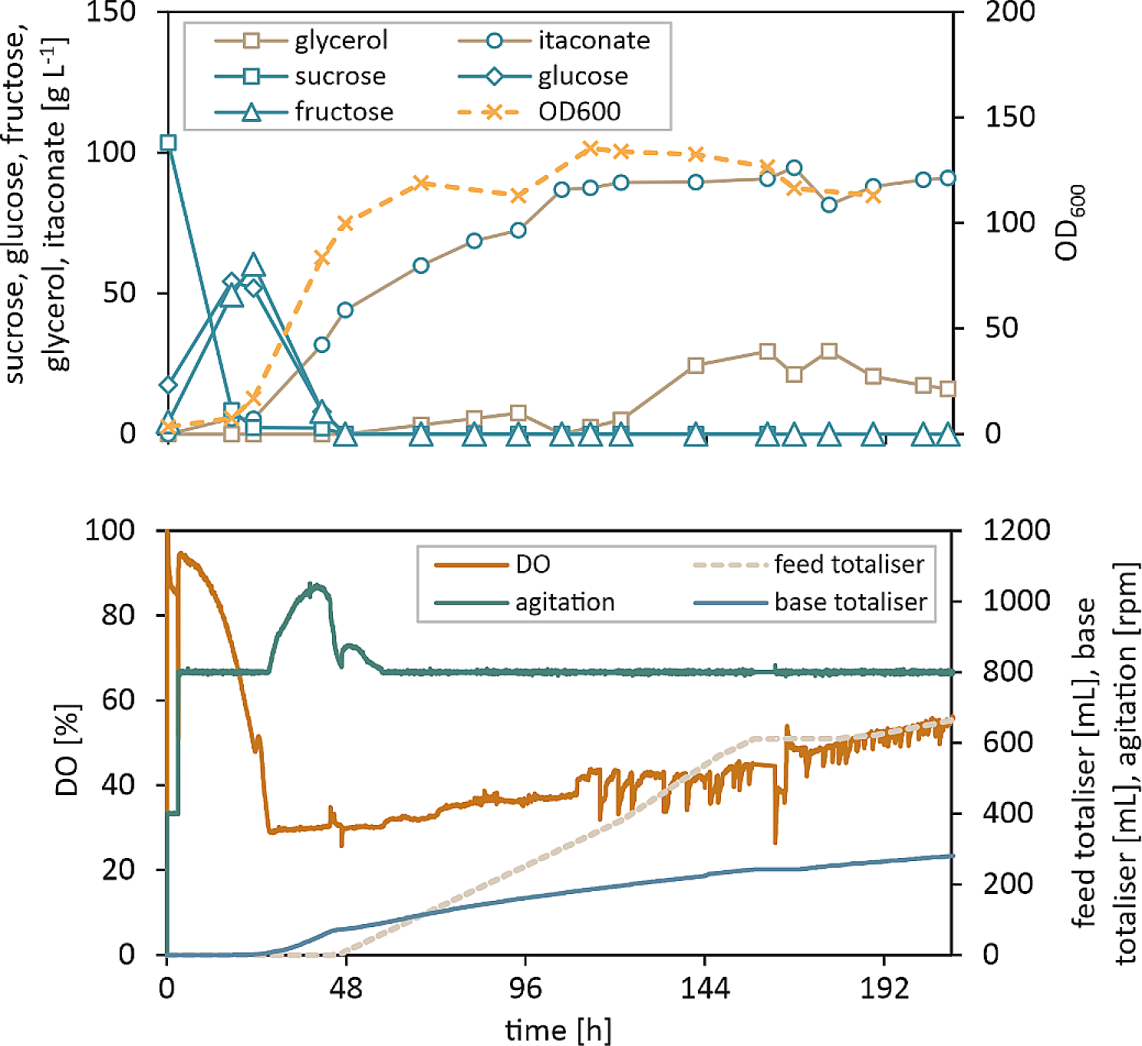
Supply of complementary feedstocks for batch and fed-batch operation. Fed-batch fermentation of *U. maydis* ori with molasses in the batch medium and crude glycerol in the feed. The cultivation was performed in a 2 L bioreactor with a filling volume of 0.9 L at 30 °C. Dissolved oxygen tension was kept > 30% and the pH was kept constant at 6.5. Samples were taken regularly and analysed via HPLC

In the following experiment, 100 g L^− 1^ sucrose equivalents from molasses were supplied only in the batch phase (Fig. 5). After 48 h, which marked the end of the batch phase, an OD_600_ of 100 was reached. The growth phase was thus 24 h shorter than that with crude glycerol, although twice as much carbon source was supplied. Once all sucrose was consumed, a feed consisting of 500 g L^− 1^ crude glycerol was started to enable growth-decoupled itaconate production. After 209 h of fermentation, a final titre of 90.93 g L^− 1^ and an overall yield of 0.33 g g^− 1^ was reached (Table 2). By supplying molasses instead of crude glycerol in the batch medium, the overall productivity was increased by 275% from 0.16 to 0.44 g L^− 1^ h^− 1^.

All KPIs reported here surpass those of a fermentation on crude glycerol (cf. Figure 4) or molasses alone [23]. A high-density fermentation on commercial glucose using the same *U. maydis* strain reached a titre of 74.9 g L^− 1^ with a yield of 0.54 g g^− 1^ at a productivity of 0.53 g L^− 1^ h^﻿−1^ [22]. This shows that the available sugars, nitrogen sources and vitamins in molasses can be used for faster biomass production and together with glycerol, which can be converted to itaconate with high yields, can lead to an overall efficient production process based on crude substrates.

### Scale-up of the dual feedstock process to 150 L

**Fig. 6:**
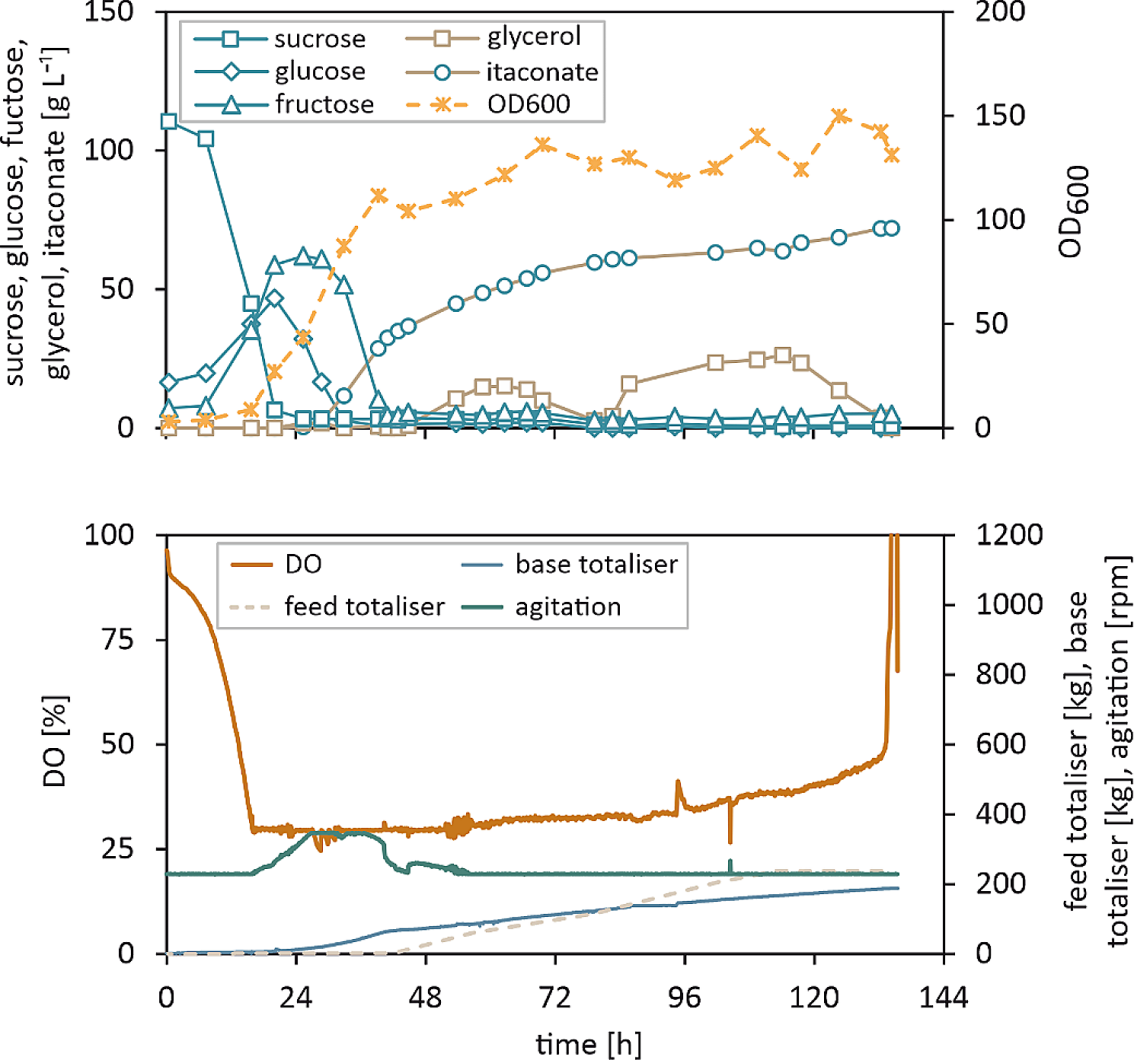
Scale-up of the two-feedstock process to 150 L. Fed-batch fermentation of *U. maydis* ori with molasses in the batch medium and 500 g L^-1^ crude glycerol feed. The cultivation was performed in a 150 L bioreactor at 30 °C. Dissolved oxygen tension was kept > 30% and the pH was kept constant at 6.5. Samples were taken regularly and analysed via HPLC

To demonstrate the industrial relevance of the process at hand, a scale-up to 150 L was performed (Fig. 6). The medium composition as well as culture conditions remained the same as in the previous fermentation at 2 L scale. Just like at the smaller scale, the sucrose from the supplied molasses was hydrolysed completely within the first 24 h and the respective monomers glucose and fructose were fully metabolised after 48 h, at which time the crude glycerol feed was started. The OD_600_ after the batch phase had reached a value of 110 and increased only marginally to a final value of 131 by the end of the fermentation. The itaconate production commenced after roughly 30 h in the batch phase and by 134 h, a final titre of 72 g L^− 1^ was reached (Table 2). While the titre was lower than that of the 2 L fermentation, the overall yield and productivity were even higher than on the smaller scale with 0.34 g g^− 1^ and 0.54 g L^− 1^ h^− 1^, respectively.

## Conclusions

In this study *U. maydis* was used for the production of itaconate from industrial waste streams. It was found that the impurities in the crude glycerol obtained from a biodiesel plant did not significantly affect the growth or itaconate production. However, it was also found that although the process had a higher yield of itaconate than with commercial glucose, the productivity was significantly lower. Therefore, two strategies were employed to further increase the performance of *U. maydis* on crude glycerol.

On the one hand, biomass growth on crude glycerol was greatly enhanced by ALE, shortening the batch phase for biomass production. However, the increase in growth rate is accompanied by a lower itaconate yield, which is likely associated with increased formation of yet-to-be identified by-products. When crude glycerol was applied under fed-batch conditions, the originally engineered strain performed better with an itaconate yield in the production phase of 0.669 g g^− 1^, representing 95% of the theoretical maximum.

As a second strategy, it was shown that the combination of different crude substrates has a positive effect on the growth rate of the cells and can increase both the itaconate titre and the overall productivity of the process. The addition of molasses, a side stream of beet sugar production containing valuable vitamins and nitrogen sources, also increased the growth rate of *U. maydis* and a final titre of 90.93 g L^− 1^ was achieved. This corresponds to a productivity increase of 275% compared to the previous fermentation which used crude glycerol during both the batch and feed phase. In addition, a scale-up to 150 L was successfully demonstrated, achieving a lower titre but higher overall yield and productivity.

In summary, it has been shown that the use of the alternative feedstock crude glycerol for the production of value-added chemicals, such as itaconate, has the potential to be a sustainable and economically viable alternative to traditional glucose-based processes, especially when combined with other sustainable feedstocks. Further research is needed to understand the cellular processes involved in glycerol metabolism, but the potential benefits make this a promising area for further investigation.

## Methods

### Chemicals and strain

All chemicals used in this study were obtained from Sigma-Aldrich (St. Louis, USA), Thermo Fisher Scientific (Waltham, USA), or VWR (Radnor, USA) and were of analytical grade. Crude glycerol was supplied by Cargill’s Bioro biodiesel refinery (Ghent, Belgium). The purity of the substrate was stated in the certificate of analysis to be 80% with major impurities including up to 10% (w w^− 1^) NaCl, up to 4% (w w^− 1^) unspecified organic matter, and 0.5% (w w^− 1^) methanol.

*Ustilago maydis* MB215 ∆*cyp3* ∆MEL ∆UA ∆*dgat* ∆P_*ria1*_::P_*etef*_ ∆*fuz7* P_*etef*_*mttA* was used for this study [22].

### Microscale cultivations

Pre-cultivations for microscale experiments were performed in 500 mL baffled shake flasks filled with 50 mL YEPS (10 g L^− 1^ yeast extract, 20 g L^− 1^ peptone, 20 g L^− 1^ sucrose). The cultivations took place in an Infors HT Multitron Pro incubator (Infors AG, Bottmingen, Switzerland) at 30 °C and 250 rpm.

All main cultivations were performed in defined modified Tabuchi medium (MTM) adapted from Geiser et al. [26]. If applicable, changes to the mentioned compositions were described in the Results and Discussion section. The standard composition of the culture medium was as follows: 2.4 g L^− 1^ NH_4_Cl, 0.4 g L^− 1^ MgSO_4_·7H_2_O, 0.01 g L^− 1^ FeSO_4_·7H_2_O, 2 g L^− 1^ KH_2_PO_4_, 0.1 M MES buffer (pH 6.5) and 1 mL L^− 1^ trace element solution. The trace element solution contained (per litre) 15 g EDTA, 4.5 g ZnSO_4_·7H_2_O, 0.84 g MnCl_2_·2H_2_O, 0.3 g CoCl_2_·6H_2_O, 0.3 g CuSO_4_·5H_2_O, 0.4 g Na_2_MoO_4_·2H_2_O, 4.5 g CaCl_2_·2H_2_O, 3 g FeSO_4_·7H_2_O, 1 g H_3_BO_3_ and 0.1 g KI. Varying carbon sources were supplied to the medium as stated for each experiment.

All microscale experiments were performed in microtiter plates of type MTP-48-BOH1 FlowerPlates® covered with F-GPRSMF32-1 gas-permeable sealing foils (all from Beckman Coulter, Baesweiler, Germany) and working volumes of 800 µL. For the cultivations, a BioLector Pro microbioreactor system (Beckman Coulter, Baesweiler, Germany) was used at 30 °C, 1400 rpm shaking frequency and relative humidity of ≥ 85%. Backscatter, pH, and DO were measured every 10 min for each cultivation well. Sacrifice samples of the cultivations were taken automatically by a Freedom Evo 200 robotic platform (Tecan Group, Männedorf, Switzerland), into which the BioLector Pro is integrated [31]. The data from these cultivations were analysed with the in-house package bletl [32, 33] and visualised using matplotlib version 3.5.2 [34].

### Adaptive laboratory evolution

ALE was performed in 500 mL shake flasks filled with 50 mL MTM containing 2.4 g L^− 1^ NH_4_Cl and 100 g L^− 1^ glycerol as the sole carbon source. The first shake flask was inoculated to an OD_600_ of 0.2 directly from cryo-cultures. The OD_600_ was checked once a day with a photometer and if it had reached a value above two, indicating that the culture had entered exponential growth, it was used to inoculate a new shake flask to an OD_600_ of 0.3 which was once again verified with a photometer. At the point of re-inoculation, a sample was taken from the now obsolete shake flask, centrifuged, the supernatant discarded, the pellet resuspended in 500 g L^− 1^ glycerol solution and stored at -80 °C. After 11 repetitive batches, the ALE was stopped and cryo stocks created, as described above, from the latest flask.

### Analytics for biomass and (by)-products

Cell dry weight (CDW) was determined by either weighing the dried cell pellets of two 1 mL samples (centrifuged at 15,000 g for 10 min, dried overnight at 80 °C) for each sample point or by using a Sartorius moisture analyser at 105 °C (Sartorius AG, Göttingen, Germany).

Concentrations of relevant metabolites in supernatants were determined by HPLC using an Agilent InfinityLab LC series Infinity II 1260 system (Agilent Technologies, Santa Clara, USA) equipped with a refractive index detector (RID). The different components were separated using an organic acid resin column (Chromatographie Service GmbH, Langerwehe, Germany) heated to 75 °C with a mobile phase of 10 mM H_2_SO_4_ running at 0.8 mL min^− 1^. Samples from the combined feedstock fermentations were separated on a Metacarb 67 H column at 35 °C with a mobile phase consisting of 2.5 mM H_2_SO_4_ running at 0.8 mL min^− 1^.

### Proteomics

For proteomic analysis, cells were grown in baffled shake flasks containing 50 mL YEPS medium (10 g L^− 1^ yeast extract, 20 g L^− 1^ peptone, 20 g L^− 1^ sucrose) for 30 h at 30 °C and 250 rpm. Subsequently this first seed stage was used to inoculate a second seed stage consisting of 50 mL MTM medium as described above with 2.4 g L^− 1^ NH_4_Cl and 50 g L^− 1^ crude glycerol. Cells were incubated until the exponential phase under the same conditions. Subsequently, 4 mL of cell suspension was spun down at maximum speed for 5 min and the supernatants were discarded.

The cell pellets were resuspended in lysis buffer containing 50 mM potassium phosphate buffer (pH 8.0), 2 mM EDTA, 2 mM 1,4-dithiothreitol (DTT), and supplemented with cOmplete protease inhibitor cocktail (1697 498, Roche Holding, Basel, Switzerland). Cell suspensions were disrupted in a Precellys System (Bertin Technologies SAS, Montigny-Le-Bretonneux, France) using MN bead tubes Type C (Macherey-Nagel, Düren, Germany) for 30 s at maximum frequency was repeated four more times. The supernatant containing protein fractions was collected and frozen at -20 °C until further analysis. Concentrations of proteins in crude extracts were measured using a Bradford assay (B6916, Sigma-Aldrich, St. Louis, USA) with BSA as standard. According to previously described methods [35], the resulting crude extracts were then applied for untargeted LC-MS/MS measurement. Briefly, a maximum of 50 µl of the crude extracts (up to 100 µg total protein) were used for tryptic digestion. Crude extracts were digested with 1 µg trypsin in a total volume of 100 µL for 5 h at 42 °C as recommended by the supplier (T7575, Sigma-Aldrich, St. Louis, USA). Peptide solutions were diluted 1:2 with MilliQ H_2_O (Millipore, Merck KGaA, Darmstadt, Germany) before LC-MS measurements. With injected sample volumes of 10 µL, protein amounts of up to 5.0 µg were applied. Peptide mixtures were separated by reversed-phase HPLC (Infinity 1260 HPLC, Agilent Technologies; column at 21 °C: 150 × 2.1 mm^2^ Ascentis Express® Peptide ES-C18 2.7 μm (53,307-U, Sigma-Aldrich, St. Louis, USA); equilibration: 3% B (12 min); gradient B: 3 − 40% (70 min), 40% (8 min), 40 − 60% (1 min), 60% (10 min); flow: 0.2 mL min^− 1^; A: 0.1% (v v^− 1^) formic acid, B: acetonitrile with 0.1% (v v^− 1^) formic acid) before ESI-MS-TOF (Q-ToF 6600 mass spectrometer, Sciex) measurements. The TripleTOF6600 was operated with CUR: 35, GS1: 50, GS2: 50, IS: 5500, TEM: 450, and DP: 120. The variable width Q1 windows were monitored in a non-scheduled manner during the elution under the above-specified parameters. SWATH window width was calculated with SWATH Variable Window Calculator_V1.0. The autosampler was set to ± 6 °C. Data acquisition and peak integration were performed using the software PeakView 2.1, while proteins were identified with the software ProteinPilot 5.1 and the Marker View software was used for *t*-test analysis (all from Sciex, Framingham, USA).

### Genome sequencing

A sequencing library with genomic DNA from the different fungal strains was prepared using the Nanopore Native Barcoding Kit 24 (SQK-NBD112-24, Oxford Nanopore Technologies, Oxford, UK) according to the manufacturer’s instructions. Sequencing was performed on an Oxford Nanopore GridION Mk1 sequencer using an R9.4.1 flow cell, which was prepared according to the manufacturer’s instructions.

Whole-genome-shotgun PCR-free libraries were constructed from 5 µg of gDNA with the Nextera XT DNA Sample Preparation Kit (Illumina, San Diego, CA, USA) according to the manufacturer’s protocol. Quality of the resulting libraries was controlled by using an Agilent 2000 Bioanalyzer with an Agilent High Sensitivity DNA Kit (Agilent Technologies, Santa Clara, CA, USA) for fragment sizes of 500–1000 bp. Paired-end sequencing was performed on the Illumina MiSeq platform (2 × 300 bp, v3 chemistry). Adapters and low-quality reads were removed by an in-house software pipeline prior to polishing.

MinKNOW (Oxford Nanopore Technologies) was used to control the run using the 72 h sequencing run protocol; base calling was performed offline using Guppy v6.3.8. The assembly was performed using flye v2.9.2. The resulting contigs were polished with Illumina short-read data using Pilon run for ten iterative cycles. BWA-MEM was used for read mapping in the first five iterations and Bowtie2 v2.3.2 in the second set of five iterations.

Gene prediction for the *U. maydis* evo strain was performed by applying GeneMark-ES 4.6.8 using default settings. Mapping of *U. maydis* ori Illumina reads on the *U. maydis* evo genome was performed using BWA-MEM. Resulting bam files were imported into ReadXplorer 2.2.3., which was used for visualization of the processed reads and detection of single-nucleotide polymorphism (SNP) in *U. maydis* evo. Minimal scores for base quality, average base quality, and average mapping quality were set to 20, and the minimum percentage of variation was 50, while the cutoff for the minimum number of varying bases was 15.

### Bioreactor cultivations on crude glycerol

For cultivation experiments in stirred tank bioreactors on glycerol as the sole carbon source a two-stage seed phase was applied. For the first stage, strains were incubated at 30 °C and 250 rpm in YEPS medium (10 g L^− 1^ yeast extract, 20 g L^− 1^ peptone, 20 g L^− 1^ sucrose) for 28 h. For the second seed stage, MTM medium as described above with 2.4 g L^− 1^ NH_4_Cl and 50 g L^− 1^ crude glycerol was used. The second seed stage lasted 67 h for *U. maydis* ori and 42 h for *U. maydis* evo. Both seed stages were performed in 500 mL baffled shake flasks with 50 mL filling volume and the cultures were inoculated to a starting OD_600_ of 0.3.

The main cultures were performed in MTM as described for the pre-cultures. The batch medium was supplied with 50 g L^− 1^ crude glycerol and the feed consisted of 600 g L^− 1^ crude glycerol. The fermentations were performed using 1.5 L DASGIP parallel bioreactor systems (Eppendorf SE, Hamburg, Germany). The fermenters were equipped with two six-blade Rushton turbines. During the cultivation the temperature was set to 30 °C. The dissolved oxygen (DO) was kept ≥ 30% automatically, by increasing the stirring rate from 400 to 1200 rpm. The aeration was fixed to 1 vvm. The pH was controlled at a value of 6.5 by the addition of 5 M NaOH and 1 M HCl. Off-gas analysis was performed using a DASGIP GA4 exhaust gas analyser (Eppendorf SE, Hamburg, Germany). Antifoam 204 (Sigma Aldrich, St. Louis, USA) was added when necessary to control foaming. Throughout the cultivation, time samples were taken from the bioreactor for offline analysis. The fermentation started with an initial volume of 800 mL. Volume changes due to feeding, pH adjusting agents and sampling were taken into account for the analysis.

### Bioreactor cultivations on combined feedstocks

For the fermentations on combined feedstocks, 500 mL liquid pre cultures were directly inoculated from YPD agar plates. The medium for the pre cultures contained (per litre) 50 g L^− 1^ glucose, 1.6 g L^− 1^ NH_4_Cl, 0.2 g L^− 1^ MgSO_4_·7H_2_O, 0.01 g L^− 1^ FeSO_4_·7H_2_O, 0.5 g L^− 1^ KH_2_PO_4_ the pH was set to 6.5 using 30 NaOH. The shake flasks were inoculated for 30 h at 30 °C and 200 rpm.

The main cultivation was performed using a 2 L DASGIP parallel bioreactor system (Eppendorf SE, Hamburg, Germany) with 0.9 L starting volume, pH kept at to 6.5 and the DO was kept ≥ 30% automatically, by increasing the stirring rate from 800 to 1200 rpm and the aeration from 0.5 to 2 vvm. The medium used for the process was as described for the pre culture, but 140 g L^− 1^ sucrose equivalents from molasses were supplied as the carbon source and 3 g L^− 1^ NH_4_Cl were added to the medium. The feed consisted of 500 g L^− 1^ crude glycerol. Antifoam 204 was added to the fermentation when necessary to control foaming.

The scale-up process at 150 L used an additional seed phase of 2 L with the same pre culture medium and cultivation conditions as described for the 2 L reaction. The shake flasks of the second seed were incubated for 43 h. The main cultivation was performed in a 150 L Frings fermenter (Heinrich Frings GmbH & Co. KG, Rheinbach, Germany) equipped with three Rushton impellers and 4 baffles in the reactor walls. The batch medium and the culture conditions were the same as for the 2 L fermentation and the feed consisted of 515 g L^− 1^ crude glycerol.

### Strain performance evaluation

KPIs from batch and fed-batch cultivation experiments with the engineered and additionally evolved strain are derived either directly from measurements or via process modelling (1.5 L fed-batch only). The process model describing fed-batch operation was formulated in Modelica using the open source editor OpenModelica [36]. In short, Monod-type kinetics were applied to describe biomass growth on two limiting medium components, namely glycerol and nitrogen. The kinetics for itaconate formation includes inhibition by nitrogen to describe the onset of production when nitrogen is limited. In addition, the formation of a previously unknown by-product is accounted for, which was necessary to close the carbon balances and properly fit the model to the data. Model parameter estimation and results visualization was performed with the in-house python-based package Estim8 (unpublished). The object-oriented framework provides a series of classes and packages for building, implementing and applying bioprocess models in the form of differential algebraic equation systems.

### Electronic supplementary material

Below is the link to the electronic supplementary material.

Supplementary Material 1

## Data Availability

All data generated or analysed during this study are included in this published article and its supplementary information files.

## Abbreviations

GC-ToF-MS: Gas chromatography coupled to time-of-flight mass spectrometry
MTM: Modified Tabuchi medium
YEPS: Yeast extract, peptone, sucrose medium
HPLC: High performance liquid chromatography
FAD: Flavin adenine dinucleotide
NAD(P)^+^: Nicotinamide adenine dinucleotide (phosphate)
ALE: Adaptive laboratory evolution
OD: Optical density
SNP: Single nucleotide polymorphism
OTR: Oxygen transfer rate
CTR: Carbon dioxide transfer rate
RQ: Respiratory quotient
CDW: Cell dry weight
DO: Dissolved oxygen
KPI: Key performance indicator
RID: Refractive index detector
