# Supplementary material for: Itaconate Production from Crude Substrates with U. maydis: Scale-up of an Industrially Relevant Bioprocess
Source: Microb Cell Fact. 2024 Jan 20;23:29. doi: 10.1186/s12934-024-02295-3 (PMC10799509; doi:10.1186/s12934-024-02295-3)
Supplement: Supplementary file 1 — Supplementary Material 1 [file 12934_2024_2295_MOESM1_ESM.docx]

**Supporting Information**

**Itaconate Production from Crude Substrates with *U. maydis*: Scale-up of an Industrially Relevant Bioprocess**

Tabea Helm^1^, Thilo Stausberg^1^, Martina Previati^2^, Philipp Ernst^1^, Bianca Klein^1^, Tobias Busche^3^, Jörn Kalinowski^4^, Daniel Wibberg^3,4^, Wolfgang Wiechert^1^, Lien Claerhout^2^, Nick Wierckx^1^, Stephan Noack^1#^


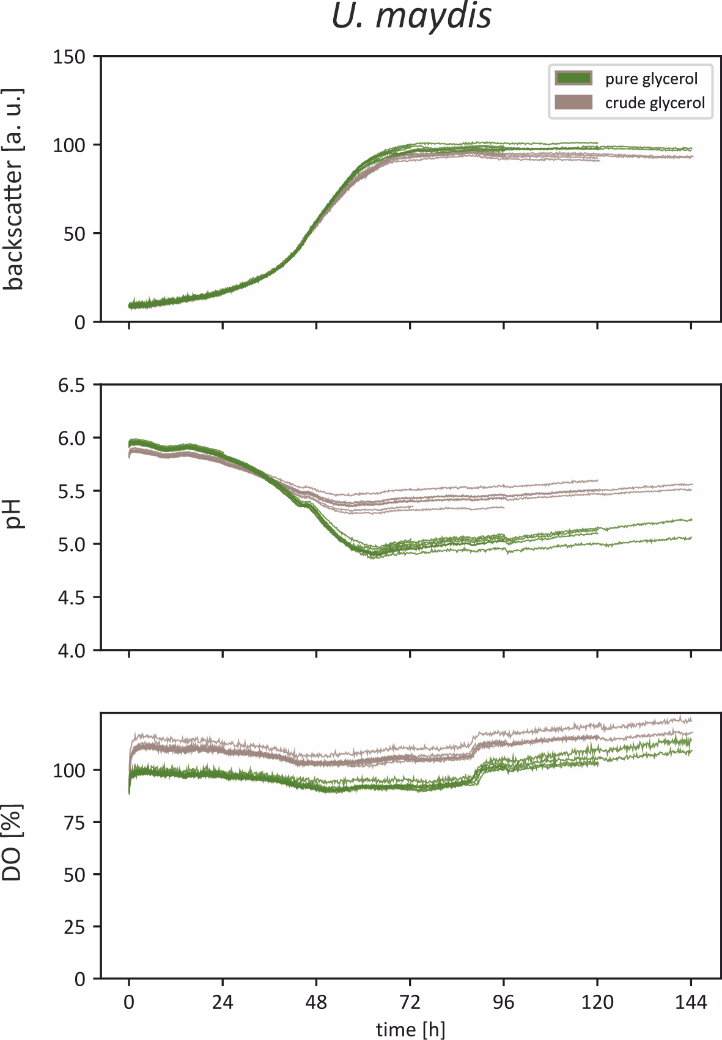


**Figure S1:** Phenotyping of U. maydis MB215 ∆cyp3 ∆MEL ∆UA ∆dgat ∆P_ria1_::P_etef_ ∆fuz7 P_etef_mttA grown in defined MTM with 50 g L^-1^ crude or pure glycerol as the sole carbon source and 2.4 g L^-1^ NH_4_Cl. The cultivation was performed in a BioLector Pro under the following conditions: 1400 rpm, 30 °C, 85 % humidity, online measurements were taken every 10 min, n = 12.


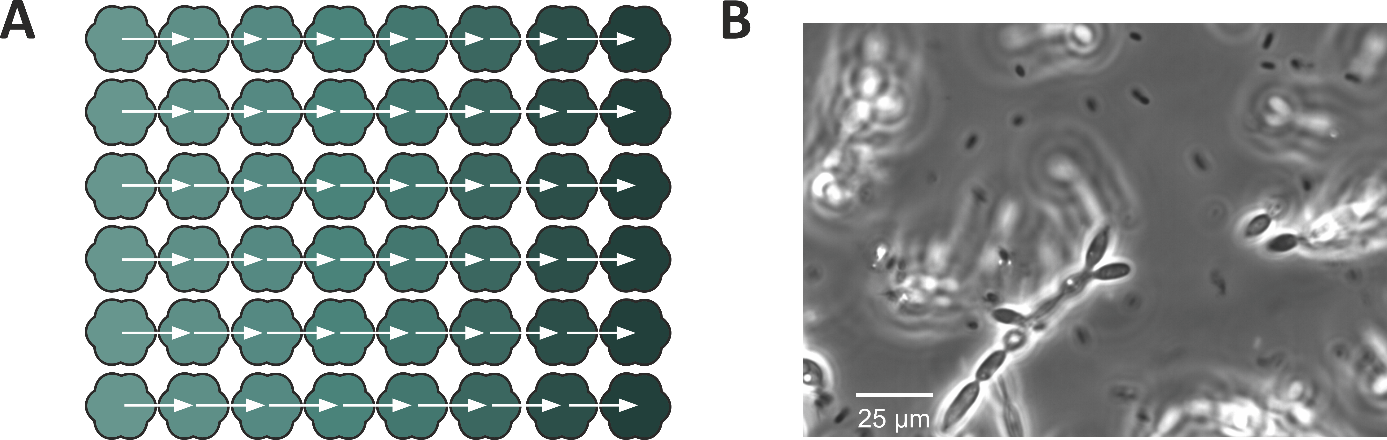


**Figure S2:** (A) Inoculation scheme of an ALE experiment performed in an automated setting in 48-well flower plates. (B) Microscopic images of U. maydis MB215 ∆cyp3 ∆MEL ∆UA ∆dgat ∆P_ria1_::P_etef_ ∆fuz7 P_etef_mttA during ALE and visible prokaryotic contamination.


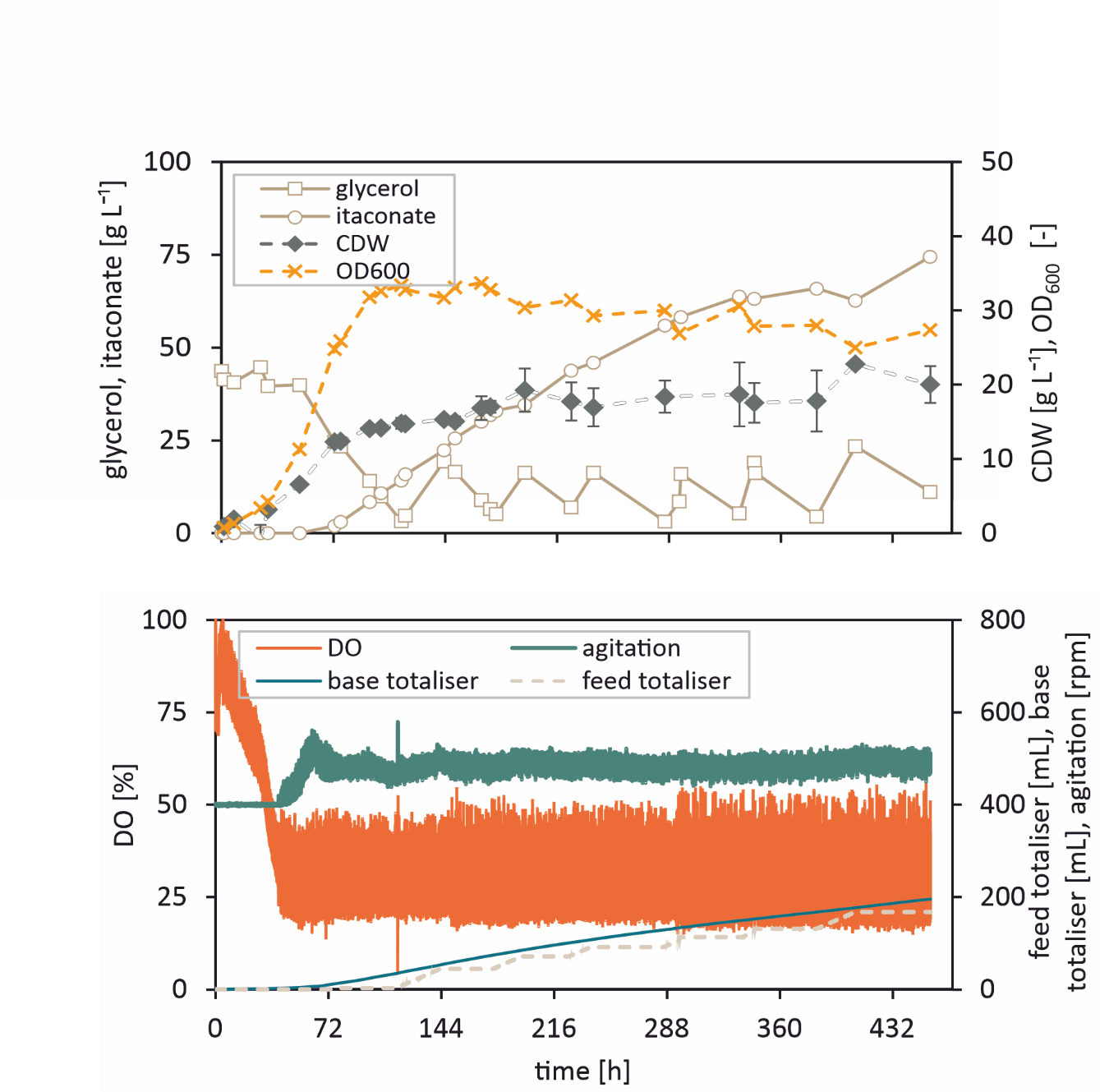


**Figure S3:** Fed-batch fermentation of U. maydis ori grown on crude glycerol as the sole carbon source in the batch medium and in the feed. The cultivation was performed in a 1.5 L DASGIP stirred tank reactor (Eppendorf AG, Hamburg, Germany) with a filling volume of 0.8 L at 30 °C. Dissolved oxygen tension was kept > 30 % and the pH was kept constant at 6.5. Samples were taken regularly and analysed via HPLC.

**
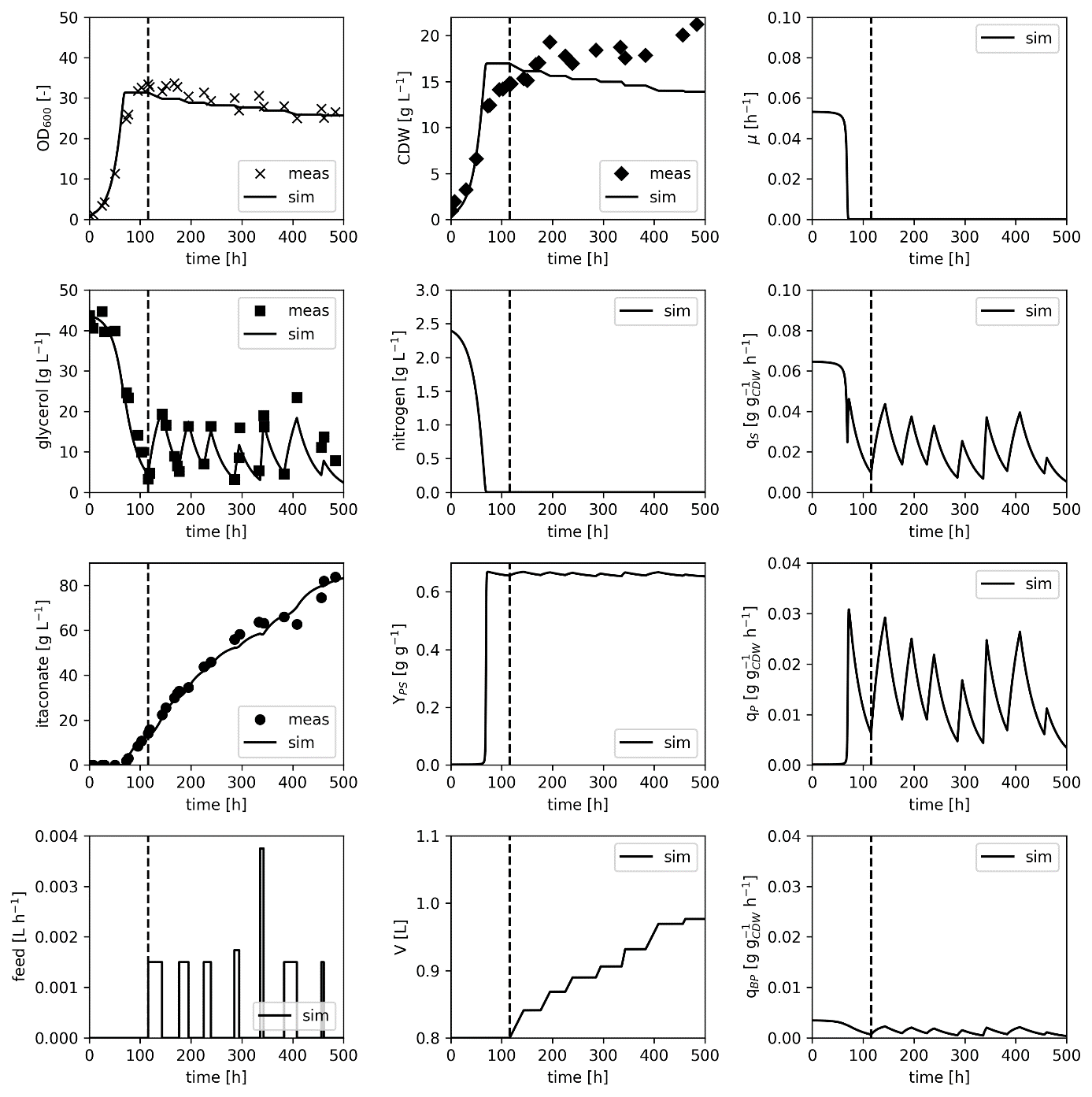
**

**Figure S4:** Results of modelling the fed-batch process with U. maydis ori grown on crude glycerol (cf. Fig. S3). The measurements for biomass (OD_600_ and CDW), glycerol, and itaconate were used to estimate the model parameters (see supplemented Modelica file). The dashed horizontal line marks the beginning of the feeding phase.

**
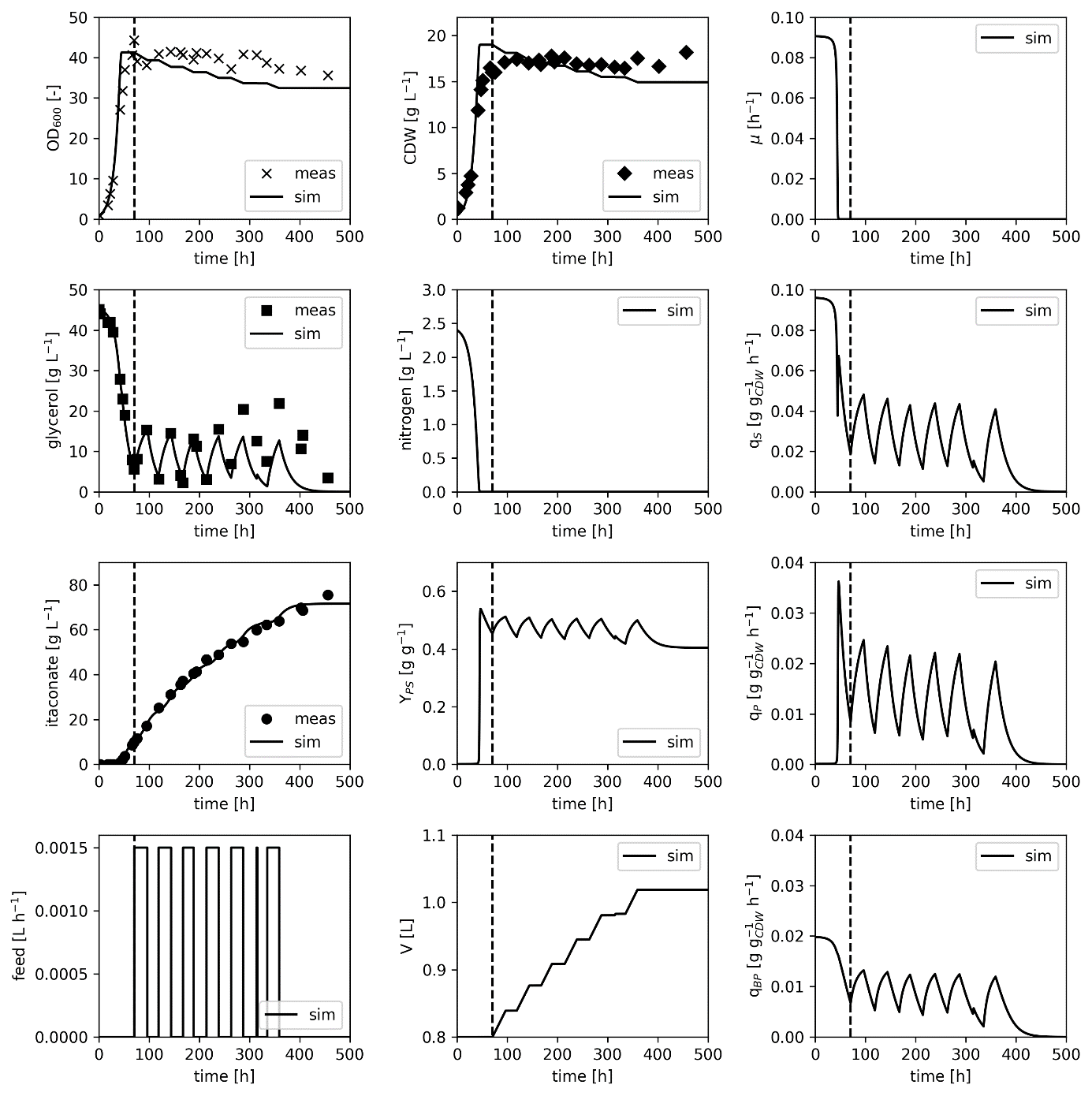
**

**Figure S5:** Results of modelling the fed-batch process with U. maydis evo grown on crude glycerol (cf. Fig. 5). The measurements for biomass (OD_600_ and CDW), glycerol, and itaconate were used to estimate the model parameters (see supplemented Modelica file). The dashed horizontal line marks the beginning of the feeding phase.
